# Prediction of cognitive impairment via deep learning trained with multi-center neuropsychological test data

**DOI:** 10.1186/s12911-019-0974-x

**Published:** 2019-11-21

**Authors:** Min Ju Kang, Sang Yun Kim, Duk L. Na, Byeong C. Kim, Dong Won Yang, Eun-Joo Kim, Hae Ri Na, Hyun Jeong Han, Jae-Hong Lee, Jong Hun Kim, Kee Hyung Park, Kyung Won Park, Seol-Heui Han, Seong Yoon Kim, Soo Jin Yoon, Bora Yoon, Sang Won Seo, So Young Moon, YoungSoon Yang, Yong S. Shim, Min Jae Baek, Jee Hyang Jeong, Seong Hye Choi, Young Chul Youn

**Affiliations:** 1 Department of Neurology, Seoul National University College of Medicine & Seoul National University Bundang Hospital, Seoul, South Korea; 2 Department of Neurology, Veterans Health Service Medical Center, Seoul, South Korea; 3 Department of Neurology, Samsung Medical Center, Sungkyunkwan University School of Medicine, Seoul, South Korea; 4 Department of Neurology, Chonnam National University Medical School, Gwangju, South Korea; 5 Department of Neurology, College of Medicine, The Catholic University of Korea, Seoul, South Korea; 6 Department of Neurology, Pusan National University Hospital, Pusan National University School of Medicine and Medical Research Institute, Busan, South Korea; 7 The Brain Fitness Center, Bobath Memorial Hospital, Seongnam, South Korea; 8 Department of Neurology, Myongji Hospital, Hanyang University College of Medicine, Goyang, South Korea; 9 Department of Neurology, University of Ulsan College of Medicine, Asan Medical Center, Seoul, South Korea; 10 Department of Neurology, Dementia Center, Ilsan Hospital, National Health Insurance Service, Goyang, South Korea; 11 Department of Neurology, College of Medicine, Gachon University Gil Hospital, Incheon, South Korea; 12 Department of Neurology, Dong-A University College of Medicine and Institute of Convergence Bio-Health, Busan, South Korea; 13 Department of Neurology, Konkuk University Medical Center, Seoul, South Korea; 14 Department of Psychiatry, University of Ulsan College of Medicine, Asan Medical Center, Seoul, South Korea; 15 Department of Neurology, Eulji University College of Medicine, Daejeon, South Korea; 16 Department of Neurology, Konyang University Hospital, College of Medicine, Konyang University, Daejeon, South Korea; 17 Department of Neurology, Ajou University School of Medicine, Suwon, South Korea; 18 Department of Neurology, Eunpyeong St. Mary’s Hospital, College of Medicine, The Catholic University of Korea, Seoul, South Korea; 19 Department of Neurology, Ewha Womans University School of Medicine, Seoul, South Korea; 20 Department of Neurology, Inha University School of Medicine, Incheon, South Korea; 21 Department of Neurology, College of Medicine, Chung-Ang University, Seoul, South Korea

**Keywords:** Machine learning, Neuropsychological test, Dementia, Mild cognitive impairment, Alzheimer’s disease

## Abstract

**Background:**

Neuropsychological tests (NPTs) are important tools for informing diagnoses of cognitive impairment (CI). However, interpreting NPTs requires specialists and is thus time-consuming. To streamline the application of NPTs in clinical settings, we developed and evaluated the accuracy of a machine learning algorithm using multi-center NPT data.

**Methods:**

Multi-center data were obtained from 14,926 formal neuropsychological assessments (Seoul Neuropsychological Screening Battery), which were classified into normal cognition (NC), mild cognitive impairment (MCI) and Alzheimer’s disease dementia (ADD). We trained a machine learning model with artificial neural network algorithm using TensorFlow (https://www.tensorflow.org) to distinguish cognitive state with the 46-variable data and measured prediction accuracies from 10 randomly selected datasets. The features of the NPT were listed in order of their contribution to the outcome using Recursive Feature Elimination.

**Results:**

The ten times mean accuracies of identifying CI (MCI and ADD) achieved by 96.66 ± 0.52% of the balanced dataset and 97.23 ± 0.32% of the clinic-based dataset, and the accuracies for predicting cognitive states (NC, MCI or ADD) were 95.49 ± 0.53 and 96.34 ± 1.03%. The sensitivity to the detection CI and MCI in the balanced dataset were 96.0 and 96.0%, and the specificity were 96.8 and 97.4%, respectively. The ‘time orientation’ and ‘3-word recall’ score of MMSE were highly ranked features in predicting CI and cognitive state. The twelve features reduced from 46 variable of NPTs with age and education had contributed to more than 90% accuracy in predicting cognitive impairment.

**Conclusions:**

The machine learning algorithm for NPTs has suggested potential use as a reference in differentiating cognitive impairment in the clinical setting.

## Background

Cognitive impairment is a spectrum that ranges from subjective cognitive decline to mild cognitive impairment (MCI) and – at its end – dementia [1]. The diagnosis of MCI and Alzheimer’s disease dementia (ADD) depends on the clinical decision by clinicians, where neuropsychological tests help inform the presence of objective cognitive impairment [2–5]. However, assessing individual cognitive states using neuropsychological test (NPT) is time-consuming, as it requires the evaluation of an extensive amount of information [6, 7]; this is in part due to the accuracy and efficiency of NPT-informed diagnosis being determined by the level of practitioner expertise.

The advent of machine learning algorithms that can analyze complex medical data may streamline the application of NPT [8, 9]. An algorithm learns the relationship between the input data (test score) and the corresponding output variables (clinical diagnosis). Once the learning process is completed, the algorithm can yield classifications or predictions when new data is inputted [10]. Several studies have applied machine learning to the differential diagnosis of dementia: Gurevich used Consortium to Establish a Registry for Alzheimer’s disease (CERAD) to identify Alzheimer’s disease (AD) among 158 subjects based on cerebral spinal fluid biomarkers and thereby achieved a classification accuracy of 89% [11]; and using a cohort of 272 subjects, Weakley et al. applied machine learning to 27 measures of NPT to yield classifications of clinical-dementia ratings. They also used machine learning to explore the configuration of measures for variable reduction and achieved an efficient predictive model using a maximum of six variables [12]. However, such investigations are among a hitherto limited effort to apply machine learning to the diagnosis and prognostic estimation of cognitive decline, and studies benefitting from large datasets are unavailable. Recently, some researchers found that some MRI and/or NPT features can be used to predict AD conversion using machine learning [13]. Although there was limited number of subjects, they used well stratified randomized dataset.

Research on screening cognitive impairment using the machine learning algorithm published by Youn et al. is similar in that it predicts cognitive impairment [14]. However, it is designed to evaluate the accuracy of a Logistic Regression algorithm based on Mini-mental status examination and simple questionnaire for screening purposes, which would be valuable in primary health care. Unlike the previous study, this work is intended to predict cognitive disorders using formal neuropsychological tests conducted by patients at hospitals, suggesting the possibility of reducing evaluators’ loads.

The Seoul Neuropsychological Screening Battery (SNSB) has been widely used for the assessment of cognitive functioning in patients with neurological disorders in Korea. The SNSB includes measures for attention, language, visuospatial function, memory and frontal executive function [15–18]. Using the SNSB, the present study applied machine learning algorithms to data on 46 variables collected from 17,139 subjects: a large set of NPT data and subjects were obtained from a well-controlled dementia cohort study [19, 20]. We thereby aimed to develop an algorithm to efficiently conduct an NPT-informed pre-reading of cognitive impairment among patients.

## Methods

The SNSB data were obtained from a study of the Clinical Research Center for Dementia of South Korea (CREDOS), memory clinics of Bundang Seoul University Hospital (BDSNUH), and Chung-Ang University Hospital (CAUH). The CREDOS study was a prospective, multi-center, hospital-based cohort study with 56 participating hospitals and was designed to assess the occurrence and risk factors of cognitive disorders [19–22]. The SNSB test was conducted by trained psychologists, and at the beginning of the CREDOS study, four workshop were held for psychiatrists or neurologists to increase the diagnostic concordance. Subjects complaining of memory lapses were clinically classified into normal cognition (NC), MCI, and AD dementia (ADD) by dementia-special clinicians based on the CREDOS criteria [14, 19, 20, 23].

**Fig. 1:**
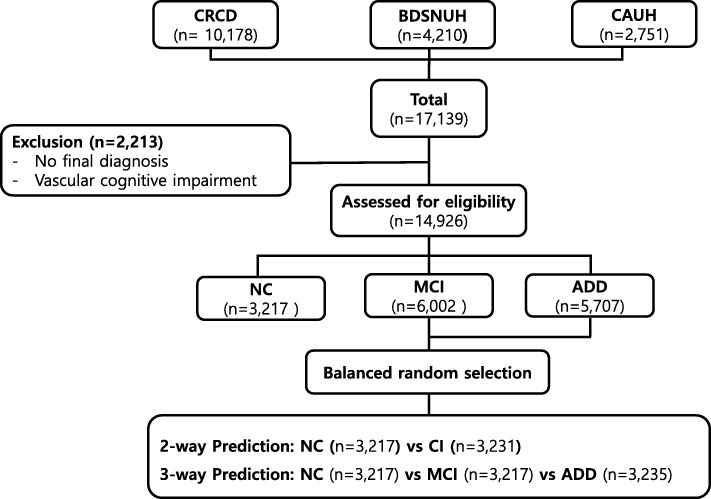
Enrollment for SNSB machine-learning analysis. CRCD, Clinical Research Center for Dementia of Korea; BDSNUH, Bungdang Seoul National University Hospital; CAUH, Chung-Ang University Hospital; NC, Normal Cognition; MCI, Mild Cognitive Impairment; ADD, Alzheimer’s Disease Dementia

A total of 17,139 subjects (10,178 from CRCD, 4210 from BDSNUH, and 2751 from CAUH) were recruited. We excluded 2213 subjects for whom no final diagnosis was available or who had severe white matter hyperintensities (deep white matter hyperintensity ≥25 mm) [19, 21, 24]; eligible subjects thus totaled to 14,926: 3217 had NC (21.55%), 6002 had MCI (40.21%), and 5707 had ADD (38.24%): “Clinic-based dataset”. The dataset was balanced by using “random.sample” method of python 3.6 through random under-sampling the majority group at nearly same to the NC: “Balanced dataset” (Fig. 1). The balanced 2-way classification dataset composed 3217 NC and 3231 CI, and 3-way classification dataset composed 3217 NC and 3217 MCI and 3235 ADD. This study was approved by the institutional review boards of the participating centers (IRB number C2012049(744)).

The features from SNSB data were 46 + 1 variables, including one target outcome (Table 1). Variables of ratio scale used raw data ​​obtained from the subjects, and ordinal scale were shown as 0, 1, 2, and 3, respectively; 0 represents subject could not perform the task at all, 1 was abnormal, 2 was borderline and 3 was normal. The variables of ordinal scale were marked with “(o)” in Table 1 . The target outcome was “clinical diagnosis” composed of participants falling within one of three diagnostic classes: NC, MCI, or ADD. We trained a machine learning algorithm using TensorFlow (https://www.tensorflow.org) to distinguish the states of the subjects based on the SNSB data [25].

**Table 1:** List of 46 features from Seoul Neuropsychological Screening Battery test

1.Education duration, 2.Age, 3.Digit span Forward, 4.Digit span Backward, 5.Letter cancellation (o), 6.Spontaneous speech fluency(o), 7.Spontaneous speech contents(o), 8.Comprehension(o), 9.Naming KBNT, 10.Finger naming(o), 11.Right left orientation(o), 12.Body part identification(o), 13.Praxis Ideomotor(o), 14.Praxis buccofacial(o), 15.Calculation total score, 16.RCFT copy score, 17.RCFT copy time, 18.SVLT recall trial1, 19.SVLT recall trial2, 20.SVLT recall trial3, 21.SVLT total recall, 22.SVLT delayed recall, 23.SVLT recognition discriminability index, 24.RCFT immediate recall, 25.RCFT delayed recall, 26.RCFT recognition discriminability index, 27.Motor impersistence(o), 28.Contrasting program(o), 29.Go No Go(o), 30.Alternating hand movement(o), 31.Alternating square and triangle(o), 32.Luria loop(o), 33.COWAT animal, 34.COWAT supermarket, 35.COWAT phonemic total score, 36.StroopTest Word reading correct, 37.StroopTest Word reading error, 38.StroopTest Color reading correct, 39.StroopTest Color reading error, 40.MMSE orientation to time, 41.MMSE orientation to place, 42.MMSE Registation, 43.MMSE attention and calculation, 44.MMSE recall, 45.MMSE language, 46.MMSE drawing, 47.Outcome	

“(o)” was marked on the features of ordinal scale. SNSB, Seoul Neuropsychological Screening Battery; BNT, Boston Naming Test; RCFT, Rey–Osterrieth Complex Figure Test; SVLT, Seoul Verbal Learning Test; COWAT, Controlled Oral Word Association Test; MMSE, Mini Mental Status Examination, RFE, Recursive Feature Elimination

### Test a. differentiate subjects with Normal cognition and cognitive impairment (Additional file 1: Table S3)

Using the two type of dataset (“clinic-based dataset” and “balance dataset”) in which the subjects were divided into the two groups of NC and cognitive impairment (CI), which included MCI and ADD, we developed an algorithm to predict for cognitive impairment (2-way classification).

The first step in modeling the algorithm requires the dataset to go through the following pre-processing steps. We imported the data formatted with ‘.csv’ and used the *train_test_split* function from scikit-learn library (https://scikit-learn.org/) to randomly split them into training and test datasets. The *train_size* was 0.75, which indicated the percentage of the data to be withheld for training; the test dataset was thus comprised of the remaining 25% of the data. Every score of features was normalized with mean and standard deviation.

The training dataset was used for further model training via TensorFlow, a commonly used open-source software library for machine learning developed by Google based on python [25]. Although it is an algorithm that differentiate subjects with CI from NC, ‘*one_hot encoding*’ was used by ‘*nb_classes = 2*’. This measure was adopted to ensure consistency when predicting NC, MCI and ADD.

This artificial neural network consisted of three layers: an input layer, an output layer, and a hidden layer. To improve the prediction, we performed Xavier method of weight initialization, and the cost was calculated via a cross entropy and minimized by means of the Adam optimizer method (Additional file 1: Table S3). The softmax classifier is used to predict the output labels. The dropout rate was 0.9, therefore 9 of 10 weights were connected to the next layer to prevent overfittings. Model training was performed with the datasets featuring all 46 variables (Table 1). Ten-fold cross-validation tests of the 2-way classifications using the training dataset was performed with *KFold* function (Additional file 1: Table S5). After validating the algorithm using 10-fold cross-validation within training datasets, we apply the algorithm 10 times on the test dataset. We thereby obtained the average of prediction accuracy, sensitivity, specificity, positive predictive value and negative predictive value of the algorithm by repeating the process 10 times which obtained from the test data.

This process was performed in both balanced dataset and clinic-based dataset.

### Test B. differentiate subjects with Normal cognition and mild cognitive impairment

The accuracy of predicting MCI was evaluated using the balanced dataset and clinic-based dataset. The previous algorithm to differentiate NC and CI was used (*A*). Training and ten-fold cross-validation test were performed also with two datasets featuring the 46 variables, and we obtained the 10 times mean prediction accuracy from the test datasets. The sensitivity, specificity, positive predictive value, and negative predictive value of the algorithm were obtained.

### Test C. differentiate subjects with normal cognition, MCI, and ADD (Additional file 1: Table S4)

The same datasets used in the Test A, but the outcome included all three outcomes (NC, MCI and ADD). These data were randomly split into training (75%) and test (25%) datasets. An artificial neural network also consisted of one input layer, one output layer, and one hidden layers. ‘*one_hot encoding*’ was used for differentiating subjects with NC, MCI and ADD by ‘nb_classes = 3’. The cost was calculated via a cross entropy and minimized by means of the Adam optimizer (Additional file 1: Table S4). The dropout rate was 0.9. We trained and tested this algorithm to predict either NC, MCI, or ADD 10 times and measured the mean accuracy of each using the test datasets. Ten-fold cross-validation tests of the 3-way classifications using the training dataset was also performed with *KFold* function (Additional file 1: Table S6).

To determine the extent to which features of the SNSB contribute to acceptable accuracy in predicting target outcome, we listed the 46 variables in order of their contribution using Recursive Feature Elimination (RFE) with a logistic regression algorithm via python 3.6 and its libraries, NumPy, and Pandas (Additional file 1: Table S2 and S3, modified from Feature Selection For Machine Learning in Python, https://machinelearningmastery.com/feature-selection-machine-learning-python/). The algorithms of Test A and C were evaluated by adding features one by one, including age and education year, until the accuracy of predicting the target outcome was greater than 90%.

## Results

**Fig. 2:**
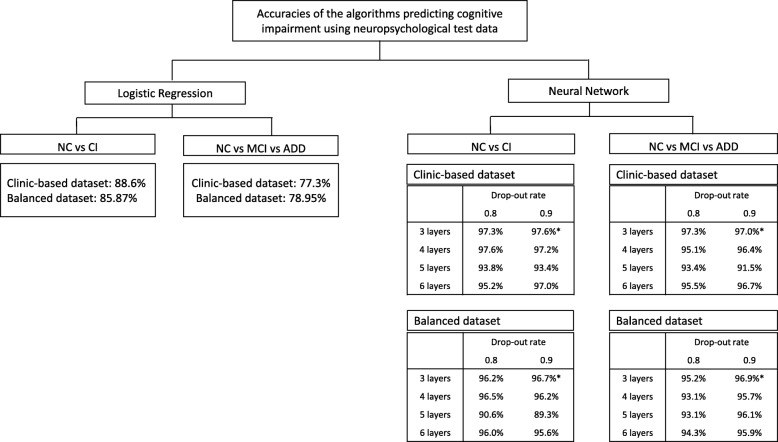
Comparison of accuracies in Logistic Regression and various layers of Neural-Network algorithm

To predict CI, MCI or ADD, the logistic regression and various layers of the neural network algorithms were compared before applying it on the SNSB features, and a 3-layer neural network with 0.9 drop-out rate was used (Fig. 2 and Additional file 1: Table S1 -S4).

Ten-fold cross-validations were performed using balanced and clinic-based training dataset. The score of cross validation in 2-way (CI vs NC) and 3-way (ADD vs MCI vs NC) classification were 96.44 ± 0.96% and 95.89 ± 0.99% in using balaced dataset; and were 97.51 ± 0.40% and 97.01 ± 0.54% in clinic based dataset (Table 2).

**Table 2:** Ten-fold cross-validation test results using balanced and clinic-based dataset

		Minimum(%)	Maximum(%)	Mean ± SD(%)
Balanced dataset	CI vs NC	95.03	97.93	96.44 ± 0.96
	MCI vs NC	94.82	97.31	96.11 ± 0.69
	ADD vs MCI vs NC	93.66	96.82	95.89 ± 0.99
Clinic-based dataset	CI vs NC	96.96	98.21	97.51 ± 0.40
	MCI vs NC	96.53	98.84	97.27 ± 0.67
	ADD vs MCI vs NC	96.34	97.86	97.01 ± 0.54

The first experiment explored whether the algorithm could accurately predict cognitive impairment from a 2-way classification dataset (CI and NC, Test A) (Table 3). The 10 times mean accuracies in identifying CI in the test datasets from the balanced dataset and the clinic-based dataset achieved by 96.66 ± 0.52% and 97.23 ± 0.32%. Their sensitivities were 91.5 and 97.4%; and the specificities were 96.8 and 95.2%. When the accuracies in predicting MCI from NC were evaluated, the mean accuracies of the balanced dataset and the clinic-based dataset were 96.60 ± 0.45 and 97.05 ± 0.38%. They showed over 95% of sensitivity and specificity.

**Table 3:** Prediction accuracy of the neural network algorithm using the neuropsychological screening test dataset

	Prediction	Number of subjects	Accuracy of 10 trials (mean ± SD%)	SE(%)	SP(%)	PPV(%)	NPV(%)	AUC
Balanced dataset	CI vs NC	3231: 3217	96.66 ± 0.52	96.0	96.8	97.0	95.8	0.964
	MCI vs NC	3217: 3217	96.60 ± 0.45	96.0	97.4	97.6	95.6	0.967
	ADD vs MCI vs NC	3235: 3217: 3217	95.49 ± 0.53					
Clinic-based dataset	CI vs NC	11,709: 3217	97.23 ± 0.32	97.4	95.2	98.6	91.3	0.963
	MCI vs NC	6002: 3217	97.05 ± 0.38	97.5	96.4	98.1	94.8	0.968
	ADD vs MCI vs NC	5707: 6002: 3217	96.34 ± 1.03					

*SD* Standard deviation, *SE* Sensitivity, *SP* Specificity, *PPV* Positive predictive value, *NPV* Negative predictive value, *AUC* Area under the curve, *CI* Cognitive impairment, *NC* Normal cognition, *MCI* Mild cognitive impairment

The last experiment (Test C) was used to assess the accuracy of the algorithm when predicting one of the three outcomes (NC, MCI or AD); the mean accuracy of the balanced dataset and the clinic-based dataset were 95.49 ± 0.53 and 96.34 ± 1.03% (Table 3).

**Fig. 3:**
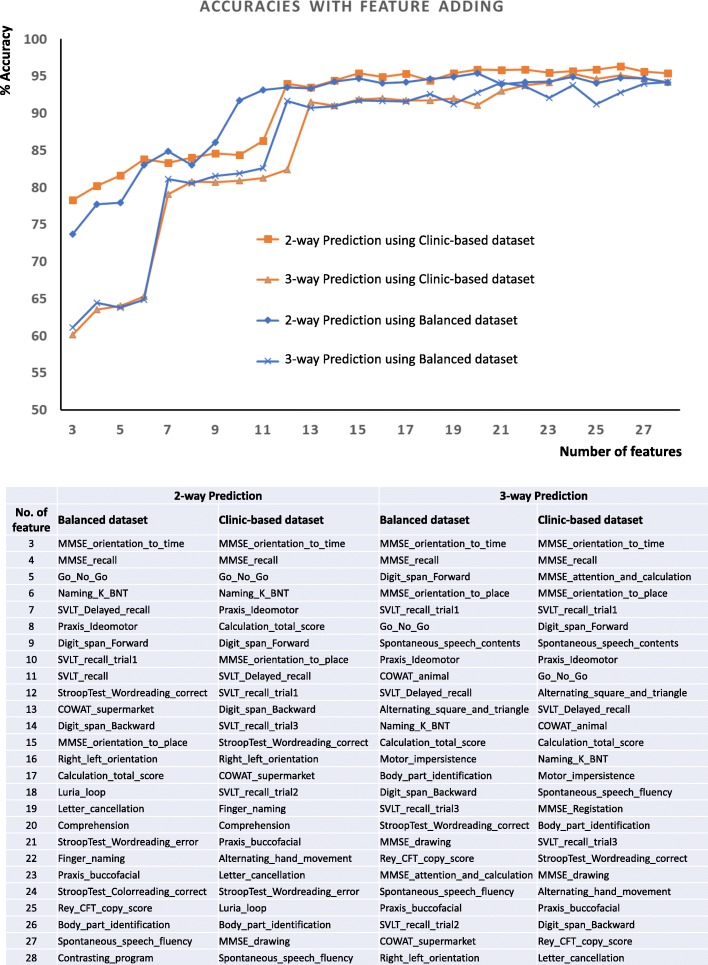
Accuracy increment with adding feature one by one

In 2-way (NC or CI) and 3-way (NC, MCI and ADD) classification, the order of 46 variables in their contribution were evaluated using the Recursive Feature Elimination (RFE). The following 2 extracted variables contributed the most to predicting the target outcome in order of ranking: ‘MMSE_orientation_to_time’ and ‘MMSE_recall’, which are memory related features. The next features contributing the outcome of the predictions were shown in Fig. 3. When ‘MMSE_orientation_to_time’, the most contributor, was added, with age and education years, the accuracy to predict cognitive impairment in the balanced dataset was 73.7%, and MCI or ADD was 61.1%. Then, an MMSE_recall was added, and the accuracy increased to 77.7 and 64.5%. When using the clinic-based dataset, the first feature was added, and the prediction of cognitive impairment was 78.3% and MCI or ADD was 60.1%. The second feature was added to increase it to 80.2 and 63.5%. However, when the number of features was 6, the accuracy was more than 80% in prediction of the cognitive impairment. As the number of features increased to 10 and 12, respectively, 2-way and 3-way classification showed more than 90% accuracy respectively (Fig. 3).

## Discussion

As an exploratory study, we first examined the logistic regression and various layers of neural network algorithms. Neural network algorithm was better than logistic regression. Among them, the 3-layer neural network algorithm was the best (Fig. 2). The accuracy of 2-way classification (NC vs CI) in the balanced dataset using the logistic regression that is commonly used for classification was 85.9%, but 3-way classification (NC vs MCI vs ADD) was only 79.0%. Compared with the logistic regression, the neural network was superior to predict the target outcome. By empirically changing the parameters one by one, we selected the parameters that showed the best performance. Particularly, when comparing 3, 4, 5, and 6-layer of the neural network, the best prediction was made in the 3-layer neural network. The dropout probability 0.8 and 0.9 were acceptable, 0.9 of which was chosen (Fig. 2), and the learning rate was 0.01. Therefore, we did supervised-learning with the 3-layer neural network in this study (Additional file 1: Table S3, S4), and found over 95% accuracy of 2-way classification and of 3-way classification (Table 3). The sensitivity and specificity of the 3-layer of neural network for the detection of CI in the balanced dataset were 96.0 and 96.8%, and MCI were 96.0 and 97.4%. The 2-way classification algorithms showed high enough sensitivity and specificity more than 85%, which is generally acceptable new biomarkers for a neurodegenerative disorder such as AD or Parkinson’s disease [26, 27], which are usuable as a reference tool [28].

There would be a concern that it may fall into a circularity problem in predicting cognitive impairment. There are two points to keep in mind when applying artificial intelligence algorithms. The first is to allow the algorithm to take over the troublesome task for human, and the second is to do better than we can do what we can’t do. The purpose of building algorithm in this study was to aid clinicians to sorting out patients with cognitive impairment from large number of cases thereby expert judges can focus on cases which require medical attention. The authors would like to have algorithms make judgments similar to those of humans when using neuropsychological tests. The algorithms only need to mimic what neuropsychologist do. However, if the aim was to make accurate diagnoses beyond human capabilities, like predicting AD by only looking at brain MRI, then the study should consider circularity issues. For more accurate diagnosis by the AI algorithm, the MRI features should not contaminate the outcome of clinical diagnosis. Since the neuropsychological tests inform the presence of objective cognitive impairment, they can necessarily influence clinical diagnosis and cannot escape circularity problem. The disease state, outcome feature of the dataset, was diagnosed finally depend on clinical decisions with considering cognitive function. While NC and CI can be classified by feature of neuropsychological test, MCI and AD dementia among patients with cognitive impairment are determined by presence of disability in daily life, which is not included as predictor in the algorithm of this study [4, 5, 28].

There are some studies having similarities in classifying patients with AD and optimizing features of neuropsychological test data to reduce the required features to predict target outcomes [9, 29]. They used the CDR score, severity of cognitive impairment, as criteria of categorization and used stratified randomization of subjects into three categories of CDR 0, 0.5 and 1. However, we classified subjects into NC, MCI and ADD by clinical decision rather than CDR, which was an different approach. Patient with CDR 0.5 could be an early stage AD or MCI, but not exclude other cause of dementia. More precisely, NC in this study was ‘subjective cognitive declines’ who visited the hospital with complaints about cognitive dysfunction and were judged normal in neuropsychological tests [30]. MCI is a condition that lies on a continuum between healthy aging and dementia [31]. Neuropsychological test, conducted by trained psychologists, is one of the information to be considered for the final diagnosis by clinicians taking into account not only neuropsychological data but also several laboratory tests and medical history obtained from the patients and their caregivers. As the algorithm lacked input from clinicians and only employed neuropsychological test data, the accuracy of predicting one out of three conditions was expected to be inevitably lower. The relatively superior accuracy of 2-way classification in small samples has also been demonstrated by prior machine-learning research [12]. It is interesting to note that using machine learning with neuropsychological data alone could distinguish accurately between MCI and ADD which requires a clinical decision. Future research can confirm the finding.

In clinic-based dataset, there were imbalances of subjects in both classifications; 2-way classification was 3217 NC vs 11,709 CI, and 3-way classification was 3217 NC vs 6002 MCI vs 5707 ADD. Although we did not perform stratification randomization, we think that it showed relatively high prediction accuracy and low variability for each trial because there was a larger dataset (Table 3). In a study with a relatively small number of subjects, stratified randomization can exclude differences by chance and can increase the reliability of the results [32]. However, we did not stratified randomization to use all possible neuropsychological data, which would be an almost real prevalence of patients visiting the hospital who want to be assessed for cognitive impairment. This study was not intended to assess neuropsychological characteristics of cognitive function nor to apply the algorithm to screening tools for community-based populations. We suggest it can be possible used as a reference when clinicians read neuropsychological tests got from hospital-based patients.

The algorithm of CI vs NC and MCI vs NC using 10–12 variables exhibited higher accuracy of prediction; there are possible implications from a dementia screening perspective. The features of the neuropsychological tests were listed in order of their contribution to the outcome using RFE (Fig. 3). Six figures with age and educational duration predicted outcomes more than 80% of the accuracy, and 12 features increased to more than 90% of the accuracy: an adequate level for machine-learning feasibility. Variable selection in machine learning is widely used to avoid data over-fit, provide faster and more effective models, and improve the accuracy of classification. Variable reduction using statistic algorithms provides the minimum subset of variables necessary for the classification model and saves time and cost for evaluation [33]. Weakley et al. conducted a study to determine the fewest number of clinical measures required for differentiating older patients with dementia from their healthy counterparts. Their results showed that as few as two to nine variables may be sufficient to obtain a clinically useful classification model [12]. It is also necessary to evaluate the value of the cognitive impairment screening test algorithm using reduced variables of the neuropsychological test.

Kang et al. compared the neuropsychological profiles between AD and mixed dementia using CREDOS dataset which target population partly overlaps with ours [34]. The current study used larger dataset and targeted to distinguish MCI and dementia in the spectrum of AD using machine learning algorithms. We tested the algorithms in the two dataset, clinic-based and balanced datasets. Although the 2-way classification (NC and MCI + ADD) was imbalanced in clinic-based dataset, the repeated trials showed low variability of accuracy and high specificity, and similar accuracies to the balanced dataset.

The present study is subject to several limitations. First, the model is only applicable to differentiate cognitive states and cannot predict the temporal stage or prognosis. Second, the dementia group only includes ADD; therefore, the model does not apply to other subtypes of dementia. Therefore more research is needed on these two respects.

The purpose of this study was to evaluate a neural network algorithm that could predict NC, MCI, and ADD from 46-features of formal neuropsychological data obtained from the hospitals. Our results indicated that 46-variable algorithm achieved acceptable accuracy, sensitivity and specificity. We also identified the order of contributions of the features that predict cognitive impairment, and approximately 12–13 from 46 features played an important role in acceptable accurate prediction.

## Conclusions

We trained and tested a machine-learning algorithm model using a large set of neuropsychological test data to distinguish between normal and cognitively impaired patients and suggest its potential use as a reference when clinicians see the neuropsychological test. Future studies are required, however, to yield an algorithm that can predict the progressor with a higher level of classification-efficiency that is capable of use in clinical settings, and can predict other causes of cognitive impairment.

## Supplementary information

**Additional file 1 Table S1.** Logistic Regression algorithm to differentiate cognitive impairment from normal cognition using the neuropsychological test dataset. **Table S2.** Logistic Regression algorithm to differentiate mild cognitive impairment and Alzheimer’s disease dementia from normal cognition using the neuropsychological test dataset. **Table S3.** Neural network algorithm to differentiate cognitive impairment from normal cognition using using the neuropsychological test dataset. **Table S4.** Neural network algorithm to differentiate mild cognitive impairment and Alzheimer’s disease dementia from normal cognition using the neuropsychological test dataset. **Table S5.** Algorithm for 10-fold cross validation of 2-way classification (cognitive impairment from normal cognition) using the neuropsychological test dataset. **Table S6.** Algorithm for 10-fold cross validation of 3-way classification (mild cognitive impairment or Alzheimer’s disease dementia from normal cognition). **Table S7.** Feature extraction with Recursive Feature Elimination from variables of the neuropsychological test (modified from Feature Selection for Machine Learning in Python, https://machinelearningmastery.com/feature-selection-machine-learning-python/).

## Data Availability

The datasets used and/or analyzed in this study are available from the CREDOS (request the data through http://public.crcd.or.kr/) and are available from the corresponding author or Seong Hye Choi, PI of CREDOS (seonghye@inha.ac.kr).

## Abbreviations

AD: Alzheimer’s disease
ADD: Alzheimer’s Disease Dementia
ADL: Activity of daily living
BDSNUH: Bungdang Seoul National University Hospital
CAUH: Chung-Ang University Hospital
CERAD: Consortium to Establish a Registry for Alzheimer’s disease
CI: Cognitive impairment
COWAT: Controlled oral word association test
CREDOS: The Clinical Research Center for Dementia of South Korea
K_MMSE: Korean version of the MiniMental Status Examination
MCI: Mild cognitive impairment
NC: Normal Cognition
NPTs: Neuropsychological tests
RCFT: Rey-Complex figure test
RFE: Recursive Feature Elimination
ROC: Receiver operator characteristic
SNSB: Seoul Neuropsychological Screening Battery
SVLT: Seoul verbal learning test
