# Supplementary material for: Prediction of cognitive impairment via deep learning trained with multi-center neuropsychological test data
Source: BMC Med Inform Decis Mak. 2019 Nov 21;19:231. doi: 10.1186/s12911-019-0974-x (PMC6873409; doi:10.1186/s12911-019-0974-x)
Supplement: Supplementary file 1 — Additional file 1 Table S1. Logistic Regression algorithm to differentiate cognitive impairment from normal cognition using the neuropsychological test dataset. Table S2. Logistic Regression algorithm to differentiate mild cognitive impairment and Alzheimer’s disease dementia from normal cognition using the neuropsychological test dataset. Table S3. Neural network algorithm to differentiate cognitive impairment from normal cognition using using the neuropsychological test dataset. Table S4. Neural network algorithm to differentiate mild cognitive impairment and Alzheimer’s disease dementia from normal cognition using the neuropsychological test dataset. Table S5. Algorithm for 10-fold cross validation of 2-way classification (cognitive impairment from normal cognition) using the neuropsychological test dataset. Table S6. Algorithm for 10-fold cross validation of 3-way classification (mild cognitive impairment or Alzheimer’s disease dementia from normal cognition). Table S7. Feature extraction with Recursive Feature Elimination from variables of the neuropsychological test (modified from Feature Selection for Machine Learning in Python, https://machinelearningmastery.com/feature-selection-machine-learning-python/). [file 12911_2019_974_MOESM1_ESM.docx]

**Supplementary Material**

**Table S1. . Logistic Regression algorithm to differentiate cognitive impairment from normal cognition using the neuropsychological test dataset.**

| import tensorflow as tf  import numpy as np  import sklearn  from sklearn.model_selection import train_test_split  tf.set_random_seed(777) # for reproducibility  # data import  csv = np.loadtxt('46feature2predDATA.csv', delimiter=',', dtype=np.float32, skiprows=1)  train, test = sklearn.model_selection.train_test_split(csv, train_size = 0.75)  # standardization of data  train_x_data = (train[:, 0:-1] - train[:, 0:-1].mean())/train[:, 0:-1].std()  train_y_data = train[:, [-1]]  test_x_data = (test[:, 0:-1] - test[:, 0:-1].mean())/test[:, 0:-1].std()  test_y_data = test[:, [-1]]  # one hot encording  nb_classes = 2 # 0 1  X = tf.placeholder(tf.float32, [None, 46])  Y = tf.placeholder(tf.int32, [None, 1]) # 1 ~ 43  Y_one_hot = tf.one_hot(Y, nb_classes) # one hot  print("one_hot", Y_one_hot)  Y_one_hot = tf.reshape(Y_one_hot, [-1, nb_classes])  print("reshape", Y_one_hot)  W = tf.Variable(tf.random_normal([46, nb_classes]), name='weight')  b = tf.Variable(tf.random_normal([nb_classes]), name='bias')  # tf.nn.softmax computes softmax activations  # softmax = exp(logits) / reduce_sum(exp(logits), dim)  logits = tf.matmul(X, W) + b  hypothesis = tf.nn.softmax(logits)  # hypothesis = tf.nn.relu(logits)  # Cross entropy cost/loss  cost_i = tf.nn.softmax_cross_entropy_with_logits(logits=logits,  labels=Y_one_hot)  cost = tf.reduce_mean(cost_i)  optimizer = tf.train.GradientDescentOptimizer(learning_rate=0.01).minimize(cost)  prediction = tf.argmax(hypothesis, 1)  correct_prediction = tf.equal(prediction, tf.argmax(Y_one_hot, 1))  accuracy = tf.reduce_mean(tf.cast(correct_prediction, tf.float32))  # Launch graph  with tf.Session() as sess:  sess.run(tf.global_variables_initializer())  for step in range(15000):  sess.run(optimizer, feed_dict={X: train_x_data, Y: train_y_data})  if step % 100 == 0:  loss, acc = sess.run([cost, accuracy], feed_dict={  X: train_x_data, Y: train_y_data})  print("Step: {:5}\tLoss: {:.3f}\tAcc: {:.2%}".format(  step, loss, acc))  # To see if we can predict  pred = sess.run(prediction, feed_dict={X: test_x_data})  # y_data: (N,1) = flatten => (N, ) matches pred.shape  t = 0  f = 0  for p, y in zip(pred, test_y_data.flatten()):  print("[{}] Prediction: {} True Y: {}".format(p == int(y), p, int(y)))  if p == int (y):  t = t + 1  else:  f = f + 1  print (t, f)  print ("The accuracy of test: ", t/(t+f)) |
| --- |

**Table S2. Logistic Regression algorithm to differentiate mild cognitive impairment and Alzheimer’s disease dementia from normal cognition using the neuropsychological test dataset.**

| import tensorflow as tf  import numpy as np  import sklearn  from sklearn.model_selection import train_test_split  tf.set_random_seed(777) # for reproducibility  # data import  csv = np.loadtxt('46feature3predDATA.csv', delimiter=',', dtype=np.float32, skiprows=1)  train, test = sklearn.model_selection.train_test_split(csv, train_size = 0.75)  # standardization of data  train_x_data = (train[:, 0:-1] - train[:, 0:-1].mean())/train[:, 0:-1].std()  train_y_data = train[:, [-1]]  test_x_data = (test[:, 0:-1] - test[:, 0:-1].mean())/test[:, 0:-1].std()  test_y_data = test[:, [-1]]  # one hot encording  nb_classes = 3 # 0 1 2  X = tf.placeholder(tf.float32, [None, 46])  Y = tf.placeholder(tf.int32, [None, 1]) # 1 ~ 46  Y_one_hot = tf.one_hot(Y, nb_classes) # one hot  print("one_hot", Y_one_hot)  Y_one_hot = tf.reshape(Y_one_hot, [-1, nb_classes])  print("reshape", Y_one_hot)  W = tf.Variable(tf.random_normal([46, nb_classes]), name='weight')  b = tf.Variable(tf.random_normal([nb_classes]), name='bias')  # tf.nn.softmax computes softmax activations  # softmax = exp(logits) / reduce_sum(exp(logits), dim)  logits = tf.matmul(X, W) + b  hypothesis = tf.nn.softmax(logits)  #hypothesis = tf.nn.relu(logits)  # Cross entropy cost/loss  cost_i = tf.nn.softmax_cross_entropy_with_logits(logits=logits, labels=Y_one_hot)  cost = tf.reduce_mean(cost_i)  optimizer = tf.train.GradientDescentOptimizer(learning_rate=0.1).minimize(cost)  prediction = tf.argmax(hypothesis, 1)  correct_prediction = tf.equal(prediction, tf.argmax(Y_one_hot, 1))  accuracy = tf.reduce_mean(tf.cast(correct_prediction, tf.float32))  # Launch graph  with tf.Session() as sess:  sess.run(tf.global_variables_initializer())  for step in range(15000):  sess.run(optimizer, feed_dict={X: train_x_data, Y: train_y_data})  if step % 100 == 0:  loss, acc = sess.run([cost, accuracy], feed_dict={  X: train_x_data, Y: train_y_data})  print("Step: {:5}\tLoss: {:.3f}\tAcc: {:.2%}".format(  step, loss, acc))  # To see if we can predict  pred = sess.run(prediction, feed_dict={X: test_x_data})  # y_data: (N,1) = flatten => (N, ) matches pred.shape  t = 0  f = 0  for p, y in zip(pred, test_y_data.flatten()):  print("[{}] Prediction: {} True Y: {}".format(p == int(y), p, int(y)))  if p == int (y):  t = t + 1  else:  f = f + 1  print (t, f)  print ("The accuracy of test: ", t/(t+f)) |
| --- |

**Table S3. Neural network algorithm to differentiate cognitive impairment from normal cognition using using the neuropsychological test dataset.**

| import tensorflow as tf  import numpy as np  import sklearn  from sklearn.model_selection import train_test_split  tf.set_random_seed(777) # for reproducibility  # data import  csv = np.loadtxt('46feature2predDATA.csv', delimiter=',', dtype=np.float32, skiprows=1)  train, test = sklearn.model_selection.train_test_split(csv, train_size = 0.75)  # standardization of data  train_x_data = (train[:, 0:-1] - train[:, 0:-1].mean())/train[:, 0:-1].std()  train_y_data = train[:, [-1]]  test_x_data = (test[:, 0:-1] - test[:, 0:-1].mean())/test[:, 0:-1].std()  test_y_data = test[:, [-1]]  # one hot encording  nb_classes = 2 # 0 1  X = tf.placeholder(tf.float32, [None, 46])  Y = tf.placeholder(tf.int32, [None, 1]) # 1 ~ 46  Y_one_hot = tf.one_hot(Y, nb_classes) # one hot  print("one_hot", Y_one_hot)  Y_one_hot = tf.reshape(Y_one_hot, [-1, nb_classes])  print("reshape", Y_one_hot)  #dropout rate  dropout_rate=tf.placeholder("float")  W1 = tf.get_variable("W1", shape=[46, 60],initializer=tf.contrib.layers.xavier_initializer(uniform=True, seed=None, dtype=tf.float32))  b1 = tf.Variable(tf.random_normal([60]))  _L1 = tf.nn.relu(tf.matmul(X, W1) + b1)  L1 = tf.nn.dropout(_L1, 0.9)    W2 = tf.get_variable("W2", shape=[60, 30],initializer=tf.contrib.layers.xavier_initializer(uniform=True, seed=None, dtype=tf.float32))  b2 = tf.Variable(tf.random_normal([30]))  _L2 = tf.nn.relu(tf.matmul(L1, W2) + b2)  L2= tf.nn.dropout(_L2, 0.9)  W3 = tf.get_variable("W3", shape=[30, nb_classes],initializer=tf.contrib.layers.xavier_initializer(uniform=True, seed=None, dtype=tf.float32))  b3 = tf.Variable(tf.random_normal([nb_classes]))  logits = tf.matmul(L2, W3) + b3  hypothesis = tf.nn.relu(logits)  # Cross entropy cost/loss  cost_i = tf.nn.softmax_cross_entropy_with_logits_v2(logits=logits, labels=Y_one_hot)  cost = tf.reduce_mean(cost_i)  optimizer = tf.train.AdamOptimizer(learning_rate=0.01).minimize(cost)  prediction = tf.argmax(hypothesis, 1)  correct_prediction = tf.equal(prediction, tf.argmax(Y_one_hot, 1))  accuracy = tf.reduce_mean(tf.cast(correct_prediction, tf.float32))  # Launch graph  with tf.Session() as sess:  sess.run(tf.global_variables_initializer())  for step in range(3000):  sess.run(optimizer, feed_dict={X: train_x_data, Y: train_y_data})  if step % 100 == 0:  loss, acc = sess.run([cost, accuracy], feed_dict={  X: train_x_data, Y: train_y_data})  print("Step: {:5}\tLoss: {:.3f}\tAcc: {:.2%}".format(  step, loss, acc))  # Let's see if we can predict  pred = sess.run(prediction, feed_dict={X: test_x_data})  # y_data: (N,1) = flatten => (N, ) matches pred.shape  t = 0  f = 0  for p, y in zip(pred, test_y_data.flatten()):  print("[{}] Prediction: {} True Y: {}".format(p == int(y), p, int(y)))  if p == int (y):  t = t + 1  else:  f = f + 1  print (t, f)  print ("The accuracy of test: ", t/(t+f)) |
| --- |

**Table S4. Neural network algorithm to differentiate mild cognitive impairment and Alzheimer’s disease dementia from normal cognition using the neuropsychological test dataset.**

| import tensorflow as tf  import numpy as np  import sklearn  from sklearn.model_selection import train_test_split  tf.set_random_seed(777) # for reproducibility  # data import  csv = np.loadtxt('46feature3predDATA.csv', delimiter=',', skiprows=1, dtype=np.float32)  train, test = sklearn.model_selection.train_test_split(csv, train_size = 0.75)  # standardization of data  train_x_data = (train[:, 0:-1] - train[:, 0:-1].mean())/train[:, 0:-1].std()  train_y_data = train[:, [-1]]  test_x_data = (test[:, 0:-1] - test[:, 0:-1].mean())/test[:, 0:-1].std()  test_y_data = test[:, [-1]]  # one hot encording  nb_classes = 3 # 0 1 2  # input placeholders  X = tf.placeholder(tf.float32, [None, 46])  Y = tf.placeholder(tf.int32, [None, 1]) # 1 ~ 46  Y_one_hot = tf.one_hot(Y, nb_classes) # one hot  print("one_hot", Y_one_hot)  Y_one_hot = tf.reshape(Y_one_hot, [-1, nb_classes])  print("reshape", Y_one_hot)  # Weight, bias and dropout for neural net layers  dropout_rate=tf.placeholder("float")  W1 = tf.get_variable("W1", shape=[46, 60],initializer=tf.contrib.layers.xavier_initializer(uniform=True, seed=None, dtype=tf.float32))  b1 = tf.Variable(tf.random_normal([60]))  _L1 = tf.nn.relu(tf.matmul(X, W1) + b1)  L1 = tf.nn.dropout(_L1, 0.9)    W2 = tf.get_variable("W2", shape=[60, 30],initializer=tf.contrib.layers.xavier_initializer(uniform=True, seed=None, dtype=tf.float32))  b2 = tf.Variable(tf.random_normal([30]))  _L2 = tf.nn.relu(tf.matmul(L1, W2) + b2)  L2= tf.nn.dropout(_L2, 0.9)  W3 = tf.get_variable("W3", shape=[30, nb_classes],initializer=tf.contrib.layers.xavier_initializer(uniform=True, seed=None, dtype=tf.float32))  b3 = tf.Variable(tf.random_normal([nb_classes]))  logits = tf.matmul(L2, W3) + b3  hypothesis = tf.nn.relu(logits)  # Cross entropy cost/loss  cost_i = tf.nn.softmax_cross_entropy_with_logits_v2(logits=logits, labels=Y_one_hot)  cost = tf.reduce_mean(cost_i)  optimizer = tf.train.AdamOptimizer(learning_rate=0.01).minimize(cost)  prediction = tf.argmax(hypothesis, 1)  correct_prediction = tf.equal(prediction, tf.argmax(Y_one_hot, 1))  accuracy = tf.reduce_mean(tf.cast(correct_prediction, tf.float32))  # Launch graph  with tf.Session() as sess:  sess.run(tf.global_variables_initializer())  for step in range(3000):  sess.run(optimizer, feed_dict={X: train_x_data, Y: train_y_data})  if step % 100 == 0:  loss, acc = sess.run([cost, accuracy], feed_dict={  X: train_x_data, Y: train_y_data})  print("Step: {:5}\tLoss: {:.3f}\tAcc: {:.2%}".format(  step, loss, acc))  # Let's see if we can predict  pred = sess.run(prediction, feed_dict={X: test_x_data})  # y_data: (N,1) = flatten => (N, ) matches pred.shape  t = 0  f = 0  for p, y in zip(pred, test_y_data.flatten()):  print("[{}] Prediction: {} True Y: {}".format(p == int(y), p, int(y)))  if p == int (y):  t = t + 1  else:  f = f + 1  print (t, f)  print ("The accuracy of test: ", t/(t+f)) |
| --- |

| **Table S5. Algorithm for 10-fold cross validation of binary prediction (cognitive impairment from normal cognition).**   \| import tensorflow as tf  import numpy as np  import sklearn  from sklearn.model_selection import KFold  from sklearn.model_selection import train_test_split  tf.set_random_seed(777) # for reproducibility  # data import  csv = np.loadtxt('CINL_equal.csv', delimiter=',', dtype=np.float32, skiprows=1)  train, test = sklearn.model_selection.train_test_split(csv, train_size = 0.75)  # standardization of data  train_x_data = (train[:, 0:-1] - train[:, 0:-1].mean())/train[:, 0:-1].std()  train_y_data = train[:, [-1]]  test_x_data = (test[:, 0:-1] - test[:, 0:-1].mean())/test[:, 0:-1].std()  test_y_data = test[:, [-1]]  # one hot encording  nb_classes = 2 # 0 1  X = tf.placeholder(tf.float32, [None, 46])  Y = tf.placeholder(tf.int32, [None, 1]) # 1 ~ 46  Y_one_hot = tf.one_hot(Y, nb_classes) # one hot  print("one_hot", Y_one_hot)  Y_one_hot = tf.reshape(Y_one_hot, [-1, nb_classes])  print("reshape", Y_one_hot)  #dropout rate  dropout_rate=tf.placeholder("float")  W1 = tf.get_variable("W1", shape=[46, 60],initializer=tf.contrib.layers.xavier_initializer(uniform=True, seed=None, dtype=tf.float32))  b1 = tf.Variable(tf.random_normal([60]))  _L1 = tf.nn.relu(tf.matmul(X, W1) + b1)  L1 = tf.nn.dropout(_L1, 0.9)    W2 = tf.get_variable("W2", shape=[60, 30],initializer=tf.contrib.layers.xavier_initializer(uniform=True, seed=None, dtype=tf.float32))  b2 = tf.Variable(tf.random_normal([30]))  _L2 = tf.nn.relu(tf.matmul(L1, W2) + b2)  L2= tf.nn.dropout(_L2, 0.9)  W3 = tf.get_variable("W3", shape=[30, nb_classes],initializer=tf.contrib.layers.xavier_initializer(uniform=True, seed=None, dtype=tf.float32))  b3 = tf.Variable(tf.random_normal([nb_classes]))  logits = tf.matmul(L2, W3) + b3  hypothesis = tf.nn.relu(logits)  # Cross entropy cost/loss  cost_i = tf.nn.softmax_cross_entropy_with_logits_v2(logits=logits, labels=Y_one_hot)  cost = tf.reduce_mean(cost_i)  optimizer = tf.train.AdamOptimizer(learning_rate=0.01).minimize(cost)  prediction = tf.argmax(hypothesis, 1)  correct_prediction = tf.equal(prediction, tf.argmax(Y_one_hot, 1))  accuracy = tf.reduce_mean(tf.cast(correct_prediction, tf.float32))  def run_train(sess, train_x, train_y):  sess.run(tf.global_variables_initializer())  for step in range(6000):  sess.run(optimizer, feed_dict={X: train_x, Y: train_y})  if step % 100 == 0:  loss, acc = sess.run([cost, accuracy], feed_dict={  X: train_x, Y: train_y})  print("Step: {:5}\tLoss: {:.3f}\tAcc: {:.2%}".format(  step, loss, acc))  def cross_validation(sess, split_size=10):  results=[]  kf=KFold(n_splits=split_size)  for train_idx, val_idx in kf.split(train_x_data, train_y_data):  train_x = train_x_data[train_idx]  train_y = train_y_data[train_idx]  val_x = train_x_data[val_idx]  val_y = train_y_data[val_idx]  run_train(sess, train_x, train_y)  results.append(sess.run(accuracy, feed_dict={X: val_x, Y: val_y}))  return results  with tf.Session() as sess:  result = cross_validation(sess)  print ("Cross-validation result: %s" % result)  print ("Test accuracy: %f" % sess.run(accuracy, feed_dict={X: test_x_data, Y: test_y_data})) \| \| --- \|   **Table S6. Algorithm for 10-fold cross validation of tertiary prediction (mild cognitive impairment or Alzheimer’s disease dementia from normal cognition)**   \| import tensorflow as tf  import numpy as np  import sklearn  from sklearn.model_selection import KFold  from sklearn.model_selection import train_test_split  tf.set_random_seed(777) # for reproducibility  # data import  csv = np.loadtxt('ADMCINL_equal.csv', delimiter=',', skiprows=1, dtype=np.float32)  train, test = sklearn.model_selection.train_test_split(csv, train_size = 0.75)  # standardization of data  train_x_data = (train[:, 0:-1] - train[:, 0:-1].mean())/train[:, 0:-1].std()  train_y_data = train[:, [-1]]  test_x_data = (test[:, 0:-1] - test[:, 0:-1].mean())/test[:, 0:-1].std()  test_y_data = test[:, [-1]]  # one hot encording  nb_classes = 3 # 0 1 2  # input placeholders  X = tf.placeholder(tf.float32, [None, 46])  Y = tf.placeholder(tf.int32, [None, 1]) # 1 ~ 46  Y_one_hot = tf.one_hot(Y, nb_classes) # one hot  print("one_hot", Y_one_hot)  Y_one_hot = tf.reshape(Y_one_hot, [-1, nb_classes])  print("reshape", Y_one_hot)  # Weight, bias and dropout for neural net layers  dropout_rate=tf.placeholder("float")  W1 = tf.get_variable("W1", shape=[46, 60],initializer=tf.contrib.layers.xavier_initializer(uniform=True, seed=None, dtype=tf.float32))  b1 = tf.Variable(tf.random_normal([60]))  _L1 = tf.nn.relu(tf.matmul(X, W1) + b1)  L1 = tf.nn.dropout(_L1, 0.9)    W2 = tf.get_variable("W2", shape=[60, 30],initializer=tf.contrib.layers.xavier_initializer(uniform=True, seed=None, dtype=tf.float32))  b2 = tf.Variable(tf.random_normal([30]))  _L2 = tf.nn.relu(tf.matmul(L1, W2) + b2)  L2= tf.nn.dropout(_L2, 0.9)  W3 = tf.get_variable("W3", shape=[30, nb_classes],initializer=tf.contrib.layers.xavier_initializer(uniform=True, seed=None, dtype=tf.float32))  b3 = tf.Variable(tf.random_normal([nb_classes]))  logits = tf.matmul(L2, W3) + b3  hypothesis = tf.nn.relu(logits)  # define cost/loss  cost_i = tf.nn.softmax_cross_entropy_with_logits_v2(logits=logits, labels=Y_one_hot)  cost = tf.reduce_mean(cost_i)  optimizer = tf.train.AdamOptimizer(learning_rate=0.01).minimize(cost)  prediction = tf.argmax(hypothesis, 1)  correct_prediction = tf.equal(prediction, tf.argmax(Y_one_hot, 1))  accuracy = tf.reduce_mean(tf.cast(correct_prediction, tf.float32))  # cross-validation  def run_train(sess, train_x, train_y):  sess.run(tf.global_variables_initializer())  for step in range(6000):  sess.run(optimizer, feed_dict={X: train_x, Y: train_y})  if step % 100 == 0:  loss, acc = sess.run([cost, accuracy], feed_dict={  X: train_x, Y: train_y})  print("Step: {:5}\tLoss: {:.3f}\tAcc: {:.2%}".format(  step, loss, acc))  def cross_validation(sess, split_size=10):  results=[]  kf=KFold(n_splits=split_size)  for train_idx, val_idx in kf.split(train_x_data, train_y_data):  train_x = train_x_data[train_idx]  train_y = train_y_data[train_idx]  val_x = train_x_data[val_idx]  val_y = train_y_data[val_idx]  run_train(sess, train_x, train_y)  results.append(sess.run(accuracy, feed_dict={X: val_x, Y: val_y}))  return results  with tf.Session() as sess:  result = cross_validation(sess)  print ("Cross-validation result: %s" % result)  print ("Test accuracy: %f" % sess.run(accuracy, feed_dict={X: test_x_data, Y: test_y_data})) \| \| --- \|   **Table S7. Feature extraction with Recursive Feature Elimination from variables of the neuropsychological test** (modified from Feature Selection for Machine Learning in Python, https://machinelearningmastery.com/feature-selection-machine-learning-python/) |
| --- | --- | --- |
| import numpy as np  from pandas import read_csv  from sklearn.feature_selection import RFE  from sklearn.linear_model import LogisticRegression  # load data  xy = read_csv('snsb_data.csv', delimiter=',', dtype=np.float32, skiprows=1)  array = xy.values  # normalization of features  x_data = (array[:, 0:-1] - array[:, 0:-1].mean())/array[:, 0:-1].std()  y_data = array[:, [-1]]  # feature extraction  model = LogisticRegression()  rfe = RFE(model, 1)  fit = rfe.fit(x_data, y_data)  print("Num Features: %d" % fit.n_features_)  print("Selected Features: %s" % fit.support_)  print("Feature Ranking: %s" % fit.ranking_) |
